# Mitigation of DMM-induced stripe patterns in synchrotron X-ray radiography through dynamic tilting

**DOI:** 10.1107/S1600577524008646

**Published:** 2024-10-25

**Authors:** Mustapha Eddah, Henning Markötter, Björn Mieller, Michael Sintschuk, Jörg Beckmann, Giovanni Bruno

**Affiliations:** a Division 8.5: X-ray Imaging Bundesanstalt für Materialforschung und -prüfung (BAM) Unter den Eichen 87 12205Berlin Germany; b Division 5.4: Advanced Multi-Materials Processing Bundesanstalt für Materialforschung und -prüfung (BAM) Unter den Eichen 87 12205Berlin Germany; Australian Synchrotron, Australia

**Keywords:** double multilayer monochromators, synchrotron X-ray imaging, signal normalization, BAMline, dynamic tilting

## Abstract

In synchrotron X-ray radiography, achieving high image resolution and an optimal signal-to-noise ratio (SNR) is crucial for the subsequent accurate image analysis. Traditional methods often struggle to balance these two parameters, especially *in situ* applications where rapid data acquisition is essential to capture specific dynamic processes. For quantitative image data analysis, using monochromatic X-rays is essential. A double multilayer monochromator (DMM) is successfully used for this aim at the BAMline, BESSY II (Helmholtz Zentrum Berlin, Germany). However, such DMMs are prone to producing an unstable horizontal stripe pattern. Such an unstable pattern renders proper signal normalization difficult and thereby causes a reduction of the SNR. We introduce a novel approach to enhance SNR while preserving resolution: dynamic tilting of the DMM. By adjusting the orientation of the DMM during the acquisition of radiographic projections, we optimize the X-ray imaging quality, thereby enhancing the SNR. The corresponding shift of the projection during this movement is corrected in post-processing. The latter correction allows a good resolution to be preserved. This dynamic tilting technique enables the homogenization of the beam profile and thereby effectively reduces noise while maintaining high resolution. We demonstrate that data captured using this proposed technique can be seamlessly integrated into the existing radiographic data workflow, as it does not need hardware modifications to classical X-ray imaging beamline setups. This facilitates further image analysis and processing using established methods.

## Abstract 2

A novel synchrotron X-ray imaging normalization technique is presented that allows for great mitigation of the presence of the double multilayer monochromator-induced stripe pattern while maintaining good resolution compared with classic flat-field correction.

## 1. Introduction

The success of synchrotron radiation sources for X-ray imaging can be explained by the increased requirements for *in situ* experiments. The high X-ray flux allows not only fast acquisitions, but also monochromatic imaging with very good spatial resolution.

Therefore, X-ray radiography and tomography using synchrotron light sources are used extensively for *in situ* probing of the inner structure of specimens in a vast variety of applications, *i.e.* biology (Zehbe *et al.*, 2012 ▸; Soares *et al.*, 2021 ▸), materials science (Arlt *et al.*, 2014 ▸, 2015 ▸; Tötzke *et al.*, 2014 ▸; Haussmann *et al.*, 2013 ▸), geosciences (Isaac *et al.*, 2016 ▸, 2018 ▸) *etc*.

For precise image analysis based on absorption, monochromatic X-rays have great advantages when it comes to quantifications of microstructures. Therefore, synchrotron beamlines often employ monochromators. Common types are double multilayer monochromators (DMMs) and double crystal monochromators (DCMs). DMMs are increasingly used for imaging instead of DCMs to provide a better balance between flux and energy resolution.

DMMs allow larger photon flux because the reflected beam coming from a DMM has a larger spectral bandwidth than a DCM [typically 1–4% versus ∼0.1% d*E*/*E* (Kastengren *et al.*, 2012 ▸; Stampanoni *et al.*, 2007 ▸; Morawe *et al.*, 2017 ▸)]. Energy resolutions of 4% are often sufficient and allow a better temporal resolution and the study of fast processes owing to the higher flux.

Furthermore, employing coherent and monochromatic radiation provides access to more sophisticated contrast modes (*e.g.*X-ray phase contrast imaging), which are especially suited for light materials.

**Figure 1:**
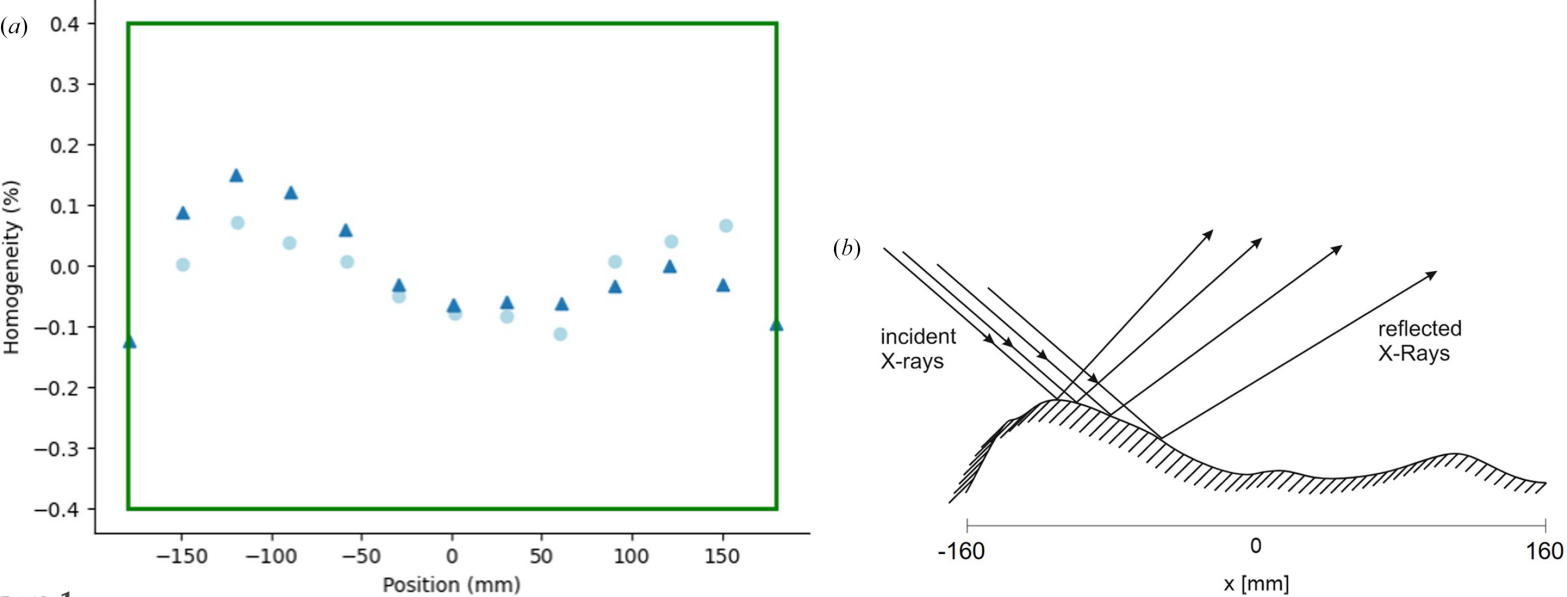
(*a*) Thickness homogeneity measurements along the sagittal direction of the W/Si DMM stripe for pre-deposition substrates (light blue circles) and final DMM stripes (dark blue triangles) at the BAMline. The lateral thickness was determined by X-ray reflectometry thickness measurements, carried out for a DMM test report by AXO Dresden GmbH. The specified optical aperture and homogeneity are marked with the green rectangle. (*b*) A simple model following the inhomogeneity measurements to explain the origin of the stripe pattern on an unnormalized image. The irregularities on the DMM surface introduce phase shifts which are then transformed to vertical intensity modulations on the detector.

However, in a DMM the beam is reflected under a very small angle in the range ∼0.1–0.5°. Consequently, the smallest surface imperfections and contaminations on the multilayer (*i.e.* deposition irregularities or impurities) lead to intensity fluctuations across the beam profile. Thickness homogeneity measurements – done via X-ray reflectometry thickness measurements – on the second W/Si multilayer mirror mounted on the BAMline at the synchrotron BESSY II (Helmholtz Zentrum Berlin, Germany) are shown in Fig. 1 ▸(*a*). The light-blue circles show measurements for the pre-substrates, and the dark-blue triangles show measurements for the final DMM stripes The thickness homogeneity measurements for both the pre-deposition substrate and the final DMM stripes roughly follow the same trend, hence reproducing inhomogeneities throughout the layers.

As shown in the model in Fig. 1 ▸(*b*), small inhomogeneities can generate intensity variations in the vertical direction (the plane containing the normal to the mirrors and the beam direction), leading to a bright–dark striped pattern on the detector downstream.

Even state-of-the-art deposition processes (Morawe, 2019 ▸) do not fully eliminate this stripe pattern (Hubert *et al.*, 2018 ▸). One recent paper links the second derivative of the wavefront to this irregular stripe pattern (Hu *et al.*, 2021 ▸).

This stripe pattern is usually dealt with via flat-field correction. This technique outputs the normalized transmission *T* by dividing the observed sample image *I*_sample_ by an image acquired without the sample in the field of view (FOV) *I*_empty_. This division is done after subtracting the dark-field signal *I*_dark_ (*i.e.* the image given by the camera without any beam) to compensate the dark current from the chip of the sCMOS camera,
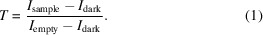
Transmission through the sample is independent of the beam and the detector profile. Inhomogeneities of the beam are not only induced by the DMM but also by the source and vacuum chamber window, which is made from 50 µm-thick Kapton foil. Dead pixels on the camera chip and dust on any part of the optical setup create constant, unremovable inhomogeneities on the captured image. For clarity, this technique will be referenced as static flat-field correction throughout this paper.

The main limit of this technique resides in the fact that it is not possible to acquire both flat-field and sample images at the same time. The sample must be removed from the FOV for the acquisition of the flat-field images for normalization. During flat-field acquisition, the sample cannot be imaged. This time loss is critical when it comes to the study of fast physical processes.

Additionally, due to thermal instabilities of the mirrors (the first being water-cooled), the beam profile is not stable throughout typical scan times. The instability of the beam also implies that, for very long experiments (*e.g.* overnight discharges of batteries, long heating procedures for ceramics *etc*.), the stripe pattern position will drift up and down throughout the experiment time. This phenomenon calls for frequent flat-field acquisitions and fewer projection image acquisitions, if one wants to achieve an acceptable image quality.

**Figure 2:**
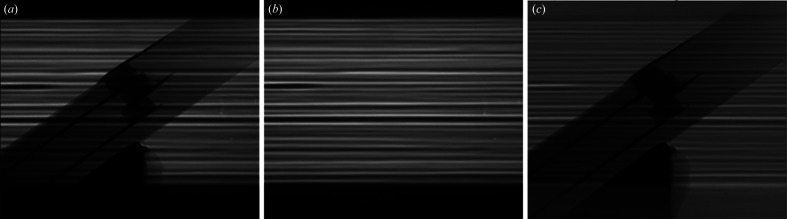
Radiographic images obtained at BAMline using 10× magnification (0.72 µm pixel size), showing the unnormalized image of (*a*) a 0.5 mm-thick LTCC test device, (*b*) a flat-field image and (*c*) the flat-field corrected image.

Fig. 2 ▸ shows radiographic images obtained during *in situ* sintering experiments performed on a low-temperature co-fired ceramic (LTCC) test device. Despite the flat-field correction, stripes are still heavily present in the final normalized image because of misalignments of the stripe pattern between (*a*) the unnormalized and (*b*) the flat-field images. Any attempts of aligning the patterns through post-experiment image treatment would enhance all other artifacts (such as those induced by impurities/scratches on the scintillator or dead pixels on the detector) and, therefore, be ineffective.

The instability of the beam profile can be attributed to a temperature gradient that builds when the first DMM mirror is heated on the top surface by the incoming white beam while being water-cooled from the bottom. Since the DMM is roughly 18 m upstream from the experiment, even the slightest change in position and orientation is enough to move the stripe pattern up and down by a few pixels (µm).

Other hardware or software based techniques are available to correct the artifacts mentioned above. Software techniques include general noise reduction in the X-ray images via wavelet Fourier filtering (Bangsgaard *et al.*, 2023 ▸) or deep-learning techniques (Grigorev & Buzmakov, 2023 ▸) whereas a hardware based approach can be random sample displacement (Hubert *et al.*, 2018 ▸) or a different cooling system to prevent multilayer vibrations (Quispe *et al.*, 2022 ▸). These approaches are powerful but require extensive processing time and manual intervention. Moreover, separating artifacts and actual sample features is very complicated when the acquisition has been done with faulty signals located on the same pixels throughout the experiment.

Here, we formulate a simple yet effective method for the reduction of this stripe pattern, based on tilting of the second multilayer mirror at the BAMline during the projection acquisition (see below). This method allows smoothing the beam profile inhomogeneities without affecting the image quality.

We also test the effectiveness of the method to reduce the DMM-induced stripe-pattern, and the effects on the resolution of the final image, comparing our method with a classic static flat-field normalization. The resolution is assessed by means of the modulation transfer functions (MTFs) and pattern analysis. To showcase the benefit of the DMM-tilt technique for image analysis, an example automatic segmentation of a multi-material sample is presented.

## 2. Experimental

### 2.1. BAMline at BESSY II

**Figure 3:**
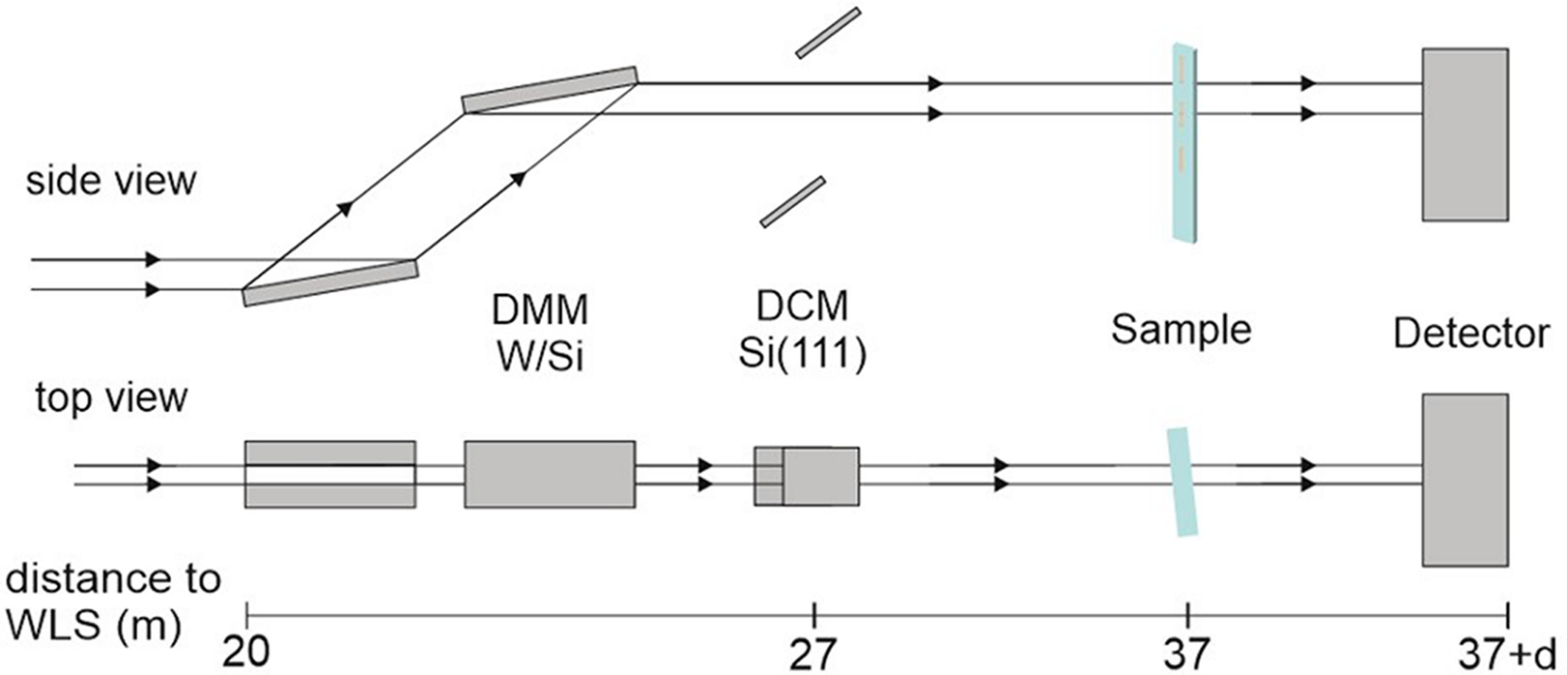
Essential layout elements of the BAMline beamline at BESSY-II: source (WLS: wavelength shifter), monochromator (DMM). The W/Si DMM has 70 nm × 3.3 nm bi-layers, resulting in Δ*E*/*E* ≃ 3–4% in a wide energy range. Not shown: filters, slit systems, windows *etc.* [for details, see Markötter *et al.* (2018 ▸)].

All experiments performed and shown here were carried out at BAMline. A detailed description of the beamline characteristics has already been provided by Markötter *et al.* (2022 ▸). Fig. 3 ▸ shows the essential layout elements of the beamline.

The DMM at BAMline consists of two Si substrates coated with three different coatings, each providing a different spectral profile. All three coatings are explained in detail by Markötter *et al.* (2022 ▸). The one used for most X-ray computed tomography (XCT) experiments is the W/Si multilayer coating, consisting of 70 bilayers, each bi-layer being 3.3 nm thick; it provides good flux while still offering an energy resolution of ∼3–4%. It must be remarked that our proposed method would work regardless of the multilayer used (Rack *et al.*, 2010 ▸, 2014 ▸).

### 2.2. Smoothing technique with height compensation

In our approach to enhance X-ray image quality, we employ a two-step correction process to address the stripe artifacts introduced by a multilayer monochromator. The image acquisition is carried out simultaneously with a defined tilt of the second mirror of the monochromator setup.

Since the second W/Si monochromator is 320 mm × 20 mm (length × width, meridional/sagittal) in size, and the incident beam angle to meet the Bragg condition is quite small (0.2–0.6°), from the white beam (∼20 mm × 20 mm) a flat beam of only a few millimetres vertical size (1–3 mm) is reflected by the DMM.

The tilt shifts the stripe pattern in the vertical direction across the whole FOV of the detector. During the tilt in this work, a total of ten projections were recorded.

**Figure 4:**
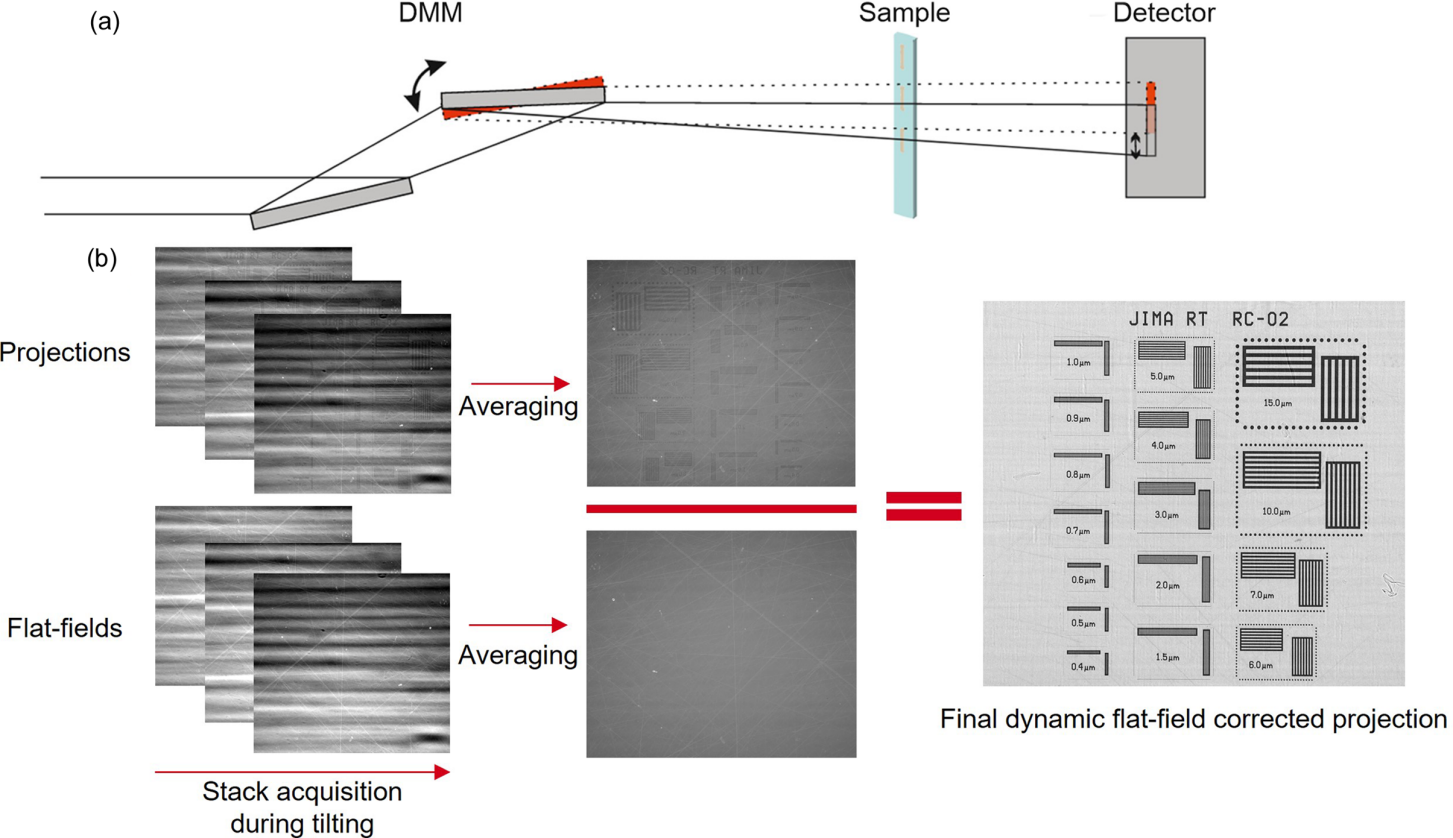
(*a*) Sketch of the tilt of the second DMM mirror and of the shift of projection in the detector. Not shown: filters, DCM, slit systems, windows *etc.* (*b*) Framework of the dynamic flat-field correction.

Due to the tilting, a vertical movement of the projection on the detector occurs, as described in Fig. 4 ▸(*a*). This displacement is a function of the sample-to-detector distance and the pixel size chosen by the user.

To counteract this vertical displacement, the user manually finds the optimal shift-per-image value between two images of the acquired stack.

Since the mirror tilting speed is constant throughout the acquisition, the vertical displacement is also constant, and the shift-per-image value can be used to correct the vertical movement for the whole acquisition.

This tilting and vertical correction are carried out both for both projections and flat-fields acquisition. The resulting ten-image stacks are then averaged and divided by each other, similarly to a static flat-field correction. The intermediate images and process are shown in Fig. 4 ▸(*b*).

Averaging these shift-corrected projections removes the stripe pattern caused by the inhomogeneities of the DMM. Through this method, we achieve a more uniform and artifact-free normalized image.

### 2.3. Assessment of the resolution

#### 2.3.1. JIMA-target RT RC-02B

A JIMA RT RC-02B (JIMA Target RT RC-02 datasheet can be found at https://jima.jp/content/pdf/rt_ct-02cata01.pdf) resolution target was imaged to determine the effectiveness of this compensation technique and to confirm that there is no loss in resolution in both the vertical and the horizontal directions. This test object is composed of various horizontal and vertical line patterns that progressively decrease in width, ranging from 15 µm to 0.4 µm, thereby increasing their spatial frequency (inverse of the pattern line width). These line patterns are made of 1 µm-thick tungsten deposited via lithography, the gaps between the lines and annotations are filled with SiO_2_ and this whole setup is fixed onto an Al plate.

Each pattern on the object is represented by a pair of dark and bright lines, typically measured in line-pairs per millimetre (lp mm^−1^).

Due to the principle of the detector design, the resolution is limited to the wavelength of the visible light emitted by the scintillator; however, owing to the beamline setup, the resolution is not limited by the X-ray wavelength, but by the wavelength of visible light emitted by the scintillator. Therefore, Abbe’s resolution limit restricts the achievable resolution to around 0.7–0.8 µm,
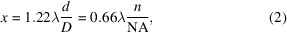
where *x* is the resolving power of our imaging system obtained from the Rayleigh criterion, *d* is the distance between the object and the lens, *D* is the aperture diameter, λ is the light wavelength, NA is the numerical aperture, and *n* is the refractive index of the medium the object is imaged in.

Since the BAMline 20× lens has an NA of 0.42 and the CdWO_4_ scintillator emission wavelength is 475 nm, we obtain a theoretical resolution limit of 0.71 µm (Markötter *et al.*, 2022 ▸).

As the lines become narrower and their spacing tighter, there comes a point where the fine-line structure can no longer be distinguished (*i.e.* there is no contrast between lines and voids). This point signifies the resolution limit of the imaging system – or its maximum spatial frequency. As a general convention, a drop in contrast below 20% in the MTF marks the achieved resolution.

This line pattern allows a direct estimation of the spatial resolution of our imaging system and a quick calculation of the MTF. The MTF estimates the frequency response of our imaging system to spatial details. High frequencies correspond to fine details in the object, and low frequencies correspond to larger features. In other words, the MTF defines the capacity of an imaging system to output the modulation of an input signal over a range of given spatial frequencies.

Radiographs were acquired at both 10× (0.72 µm pixel size, 1.05 µm Rayleigh resolution limit) and 20× magnification (0.36 µm pixel size, 0.71 µm Rayleigh resolution limit). The 10× magnification was chosen to confirm the effectiveness of our technique in eliminating stripe artifacts, while the 20× magnification was chosen to ensure that this normalization technique does not compromise the image resolution.

The radiographs of the JIMA target were first quickly screened for quality using *Fiji* (Schindelin *et al.*, 2012 ▸). For a more thorough analysis [*e.g.* contrast-to-noise ratio (CNR)/signal-to-noise ratio (SNR) calculation and MTF plotting], we developed a Python code based on the *Pyqtgraph* library (https://www.pyqtgraph.org/), which allowed us to analyze a large number of images simultaneously. The plotted profiles were corrected with a linear baseline to account for gray value gradients.

The 20× scans were normalized using static flat-field correction, and our DMM-dynamic normalization was carried out with and without height compensation in order to assess the importance of proper vertical shifting of the acquired projections, resulting in three different normalization approaches.

Subsequently, the MTF profiles are computed by calculating the modulation intensities for all available line patterns, both vertical and horizontal. The signal modulation is calculated as *M* = (*a* − *b*)/(*a* + *b*), with the parameters defined in Fig. 6.

It is emphasized that this definition is only applicable to line structures with equivalent gap and line widths. Uncertainty on the JIMA-derived MTF considers the specified manufacturing tolerance of the line widths, ranging from 8% to 10% depending on structure size, and repeatability uncertainty on the MTF value (averaged over five MTF measurements per line structure).

The metrics CNR and SNR are used to analyze the effectiveness of our stripe-smoothing method:
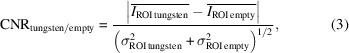

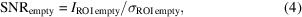
where 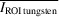 and 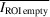 are the mean of pixel gray values in vertical ‘empty’ regions (also regions of interest, ROIs) of the image (*i.e.* ROIs where the stripe pattern is most visible without sample structure), and σ_ROItungsten_ and σ_ROIempty_ are the standard deviations in the same ROIs.

#### 2.3.2. Ceramic test structures

Modern devices in microelectronics and microsystems technology demand an increasing degree of integration and miniaturization. Especially in the already highly developed ceramic multilayer technology, the demand for high-frequency components (*e.g.* for current 5G technology and millimetre-wave applications) requires a further reduction of structural sizes such as layer thickness and spacing of metallic conductors (Schindelin *et al.*, 2012 ▸; Matz *et al.*, 2020 ▸).

LTCC devices are self-contained, ceramic microelectronic devices that incorporate conductive or electric materials within a ceramic matrix. These components are known for their high performance, density and stable interconnections, making them ideal for a wide array of applications, from MEMS (microelectromechanical systems) sensors and actuators to 3D fluidic integration for sensors and microreactors (Matz *et al.*, 2020 ▸; Bandyopadhyay & Heer, 2018 ▸; Zhao *et al.*, 2018 ▸; Feller & Partsch, 2021 ▸).

In addition to the ceramic component (Al_2_O_3_) and the residual glass phase, the final microstructure also contains various crystalline phases, for example anorthite or celsian (Beate Capraro *et al.*, 2022 ▸). Further components of the microstructure are pores in both the metal and the glass ceramic, as well as glassy components in the metal structures.

The radiographic and tomographic reconstruction of such materials is challenging due to the complex microstructure and presence of both strongly absorbing parts [*i.e.* the metallic screen-prints and vertical interconnect accesses (VIAs)] and weakly absorbing parts (*i.e.* the ceramic components and pores).

The presence of both high- and low-absorbing materials in the sample makes the quantifications of microstructure especially challenging if DMM-induced stripe patterns are present. In fact, a clean reconstruction and/or image analysis requires consistent gray level response throughout the sample, to identify cracks, holes and misalignments between low- and high-absorbing materials.

To verify whether the DMM-tilting method is effective, an LTCC device comprised of screen-printed Ag lines on CT708 sheets, inter-connected by Ag/Pd VIAs, was imaged. This design, referred to as a daisy chain, is frequently used to assess the precision and accuracy of the LTCC manufacturing process. A simple electric continuity test is sufficient to confirm that all LTCC layers (and VIAs) are aligned.

## 3. Results

### 3.1. JIMA-target

#### 3.1.1. 10× magnification

**Figure 5:**
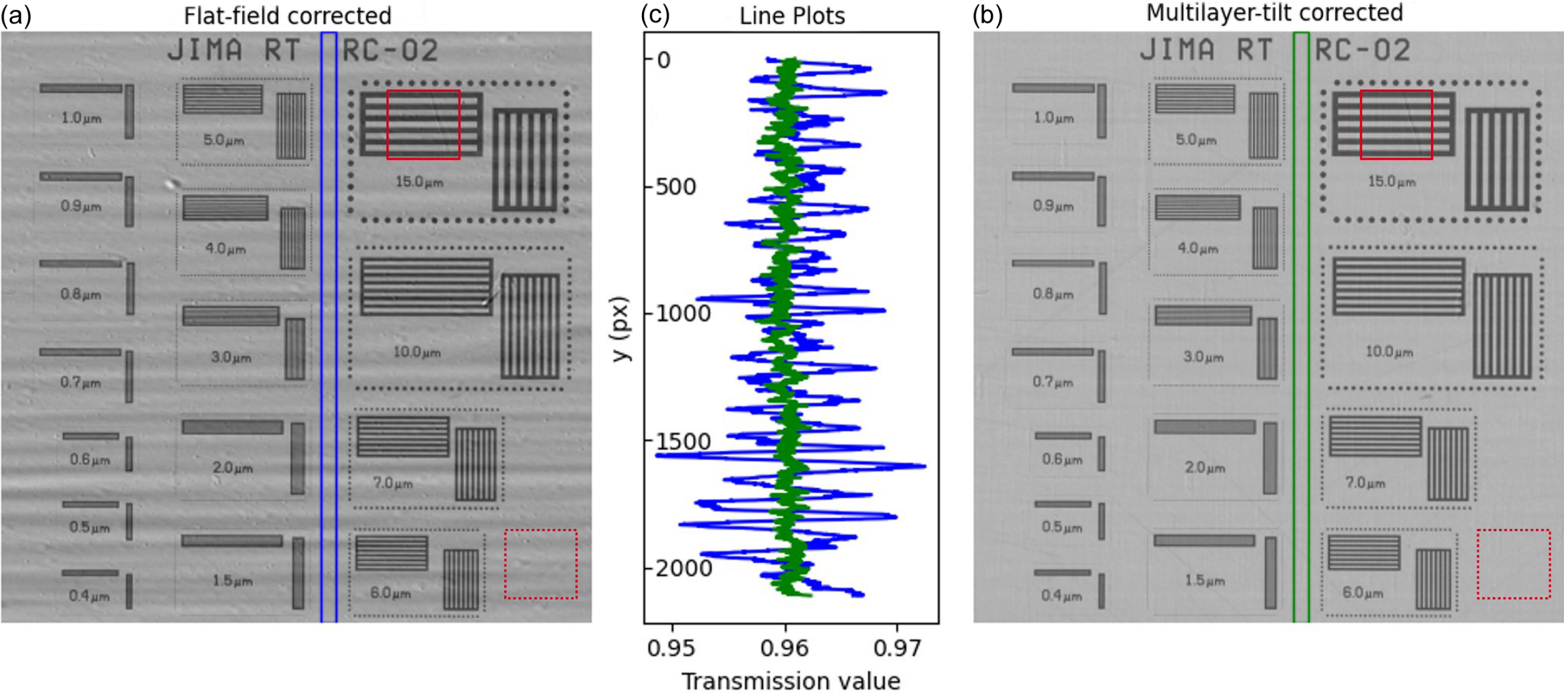
(*a*) Static flat-field correction *I*_static−FF_ of 10× radiography of the JIMA RT RC-02 target. (*b*) Dynamic flat-field correction *I*_DMM − FF_ of the same FOV and (*c*) transmission values plotted through both images in the same empty region (averaged over a 50 pixel line width). Both radiographs were taken at 25 keV and at a distance of 15 mm from the detector. The red rectangles in (*a*) and (*b*) show the ROIs used for CNR/SNR calculations, centered on tungsten lines (solid rectangle) and on the background (dashed lines).

Figs. 5 ▸(*a*) and 5 ▸(*b*) show radiographs of the JIMA RT RC-02 taken at a 10× magnification (0.7 µm pixel) with a detector-to-sample distance of 15 mm and a beam energy of 25 keV.

*I*_static−FF_ was normalized using flat-field correction while *I*_DMM_ was normalized using our multilayer-tilting normalization. Ten projections were used for both techniques, with an exposure time of 300 ms per projection.

The suppression of the stripes in the *I*_DMM_ image is clearly visible across the entire FOV. This is made particularly evident by the plot of intensity profiles along the vertical direction, shown in Fig. 5 ▸(*c*). In Fig. 5 ▸(*c*), the transmission is heavily affected by the presence of the stripe pattern in the static normalized image.

The CNR has been evaluated using the standard deviation and the mean of ROIs of the same size centered both in the background (SiO_2_) of the JIMA target and in the tungsten lines, these ROIs are shown in Fig. 5 ▸. The SNR has been evaluated using ROIs centered on the background (SiO_2_) of the JIMA target.

CNR, SNR and standard deviation values of the plotted background values of Fig. 5(*c*) ▸ are given in Table 1 ▸.

Upon use of our method, the SNR and CNR are improved by 55% and 38%, respectively, while the standard deviation has improved by a factor of five.

#### 3.1.2. 20× magnification

**Figure 6:**
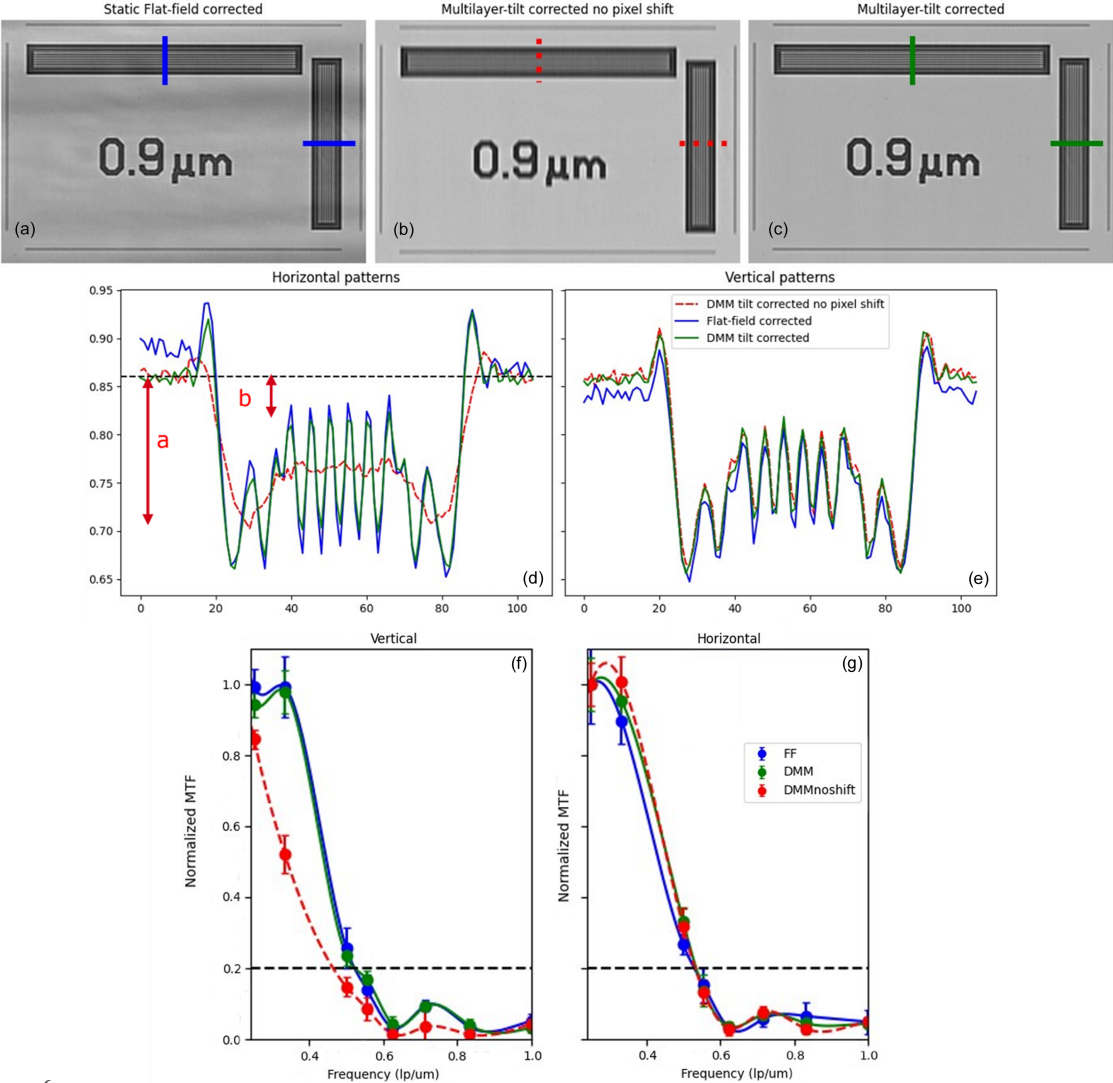
Panels in the upper row show radiographs normalized through (*a*) static flat-field correction, (*b*) multilayer tilting without shift compensation, (*c*) multilayer tilting with shift compensation. (*d*) and (*e*) Profile plots along the corresponding colored horizontal and vertical lines on panels (*a*)–(*c*). The dotted black horizontal line represents the baseline used for MTF calculation, with values *a* and *b* used highlighted in red. (*f*) Vertical and (*g*) horizontal MTF computed from the 20× JIMA target radiographs for all normalization methods.

Figs. 6 ▸(*a*)–6 ▸(*c*) show radiographs taken with a 20× magnification, centered around the 0.9 µm pattern size, with a detector-to-sample distance of 15 mm and a 15 keV energy.

Fig. 6 ▸(*a*) shows a normalized projection using static flat-field correction, *I*_static−FF_. The stripe pattern is still heavily present despite the *I*_empty_ projection containing the same pattern.

Figs. 6 ▸(*b*) and 6 ▸(*c*) show the same ROI normalized using our technique without (*I*_DMM − noshift_) and with shift compensation (*I*_DMM_), respectively. The horizontal stripe pattern is completely blurred without the shift compensation, and the details are only preserved with the shift-compensation *I*_DMM_.

The *I*_static−FF_ and *I*_DMM_ show the same level of detail in both the horizontal and the vertical patterns, whereas the *I*_DMM−noshift_ projection sees a substantial degradation in image quality for the horizontal line pattern, hence confirming the need for shift-compensation in the final processing method.

Figs. 6 ▸(*d*)–6 ▸(*e*) show the profile plots along the corresponding colored lines in Figs. 6 ▸(*a*)–6 ▸(*c*), perpendicular to the horizontal and vertical line patterns. The *I*_DMM_ profile plot agrees with the *I*_static−FF_ plot, both horizontally and vertically, whereas the *I*_DMM−noshift_ shows very little modulation for vertical JIMA line patterns as noticed in the images already. Although this means that in terms of resolution and contrast the static and dynamic approaches are somewhat equivalent in the case of strongly absorbing materials, it also means that blurring the images by tilting the DMM does not introduce any loss of contrast or resolution. However, this blurring becomes significant in the case of weakly absorbing materials, where image segmentation would become nearly impossible in the presence of intensity stripes (*i.e.* without dynamic correction).

Additionally, modular transfer functions (MTFs) were calculated from the normalized projections generated by all three techniques. Figs. 6 ▸(*f*)–6 ▸(*g*) show the computed horizontal and vertical MTFs for line-patterns ranging from 2 µm to 0.5 µm width. Horizontal patterns are used to determine resolution power in the vertical direction and vice versa. For each line pattern five ROIs from different locations were taken to assess statistical inaccuracies. Error bars in Figs. 6 ▸(*f*) and 6 ▸(*g*) indicate the variations.

Of course, the *I*_DMM−noshift_ MTF shows very poor resolution power in the horizontal lines, since the projection shift is not compensated in that direction, hence blurring the horizontal patterns on the ROI. The MTFs computed from JIMA patterns in Fig. 6 ▸ show that the improvement of the SNR and CNR (Table 1 ▸) does not come at the cost of lower resolution, since with proper alignment, no information is lost by averaging the final stack of projections.

*I*_static−FF_ and *I*_DMM_ show the same characteristic phase contrast MTF shape, with the highest MTF value not necessarily corresponding to the lowest frequency. This type of pattern is characteristic of the effect of the phase contrast in the image (*i.e.* interference fringes at the edges). These fringes correspond to the overshoot seen in Fig. 6 ▸(*c*) and cause the additional peak in the MTF plot, centered around the 0.7 lp µm^−1^ frequency. Havariyoun *et al.* (2023 ▸) explain this phenomenon quite well, and the reader is referred to such work for details.

The uncertainties for the vertical *I*_static−FF_ MTF are generally higher than that of the horizontal one, since the perturbing stripe pattern is horizontal, hence causing high standard deviation in the measured intensities.

### 3.2. LTCC daisy chain: use case

Sintered LTCC daisy chain structures were imaged *ex situ* using both regular flat-field correction and our multilayer-tilting technique to validate the technique on technologically relevant samples. This experiment was also carried out at the BAMline beamline, and the experimental parameters were the same as for the JIMA target described above. However, we had to increase the photon energy to 40 keV to obtain sufficient transmission through the sample.

**Figure 7:**
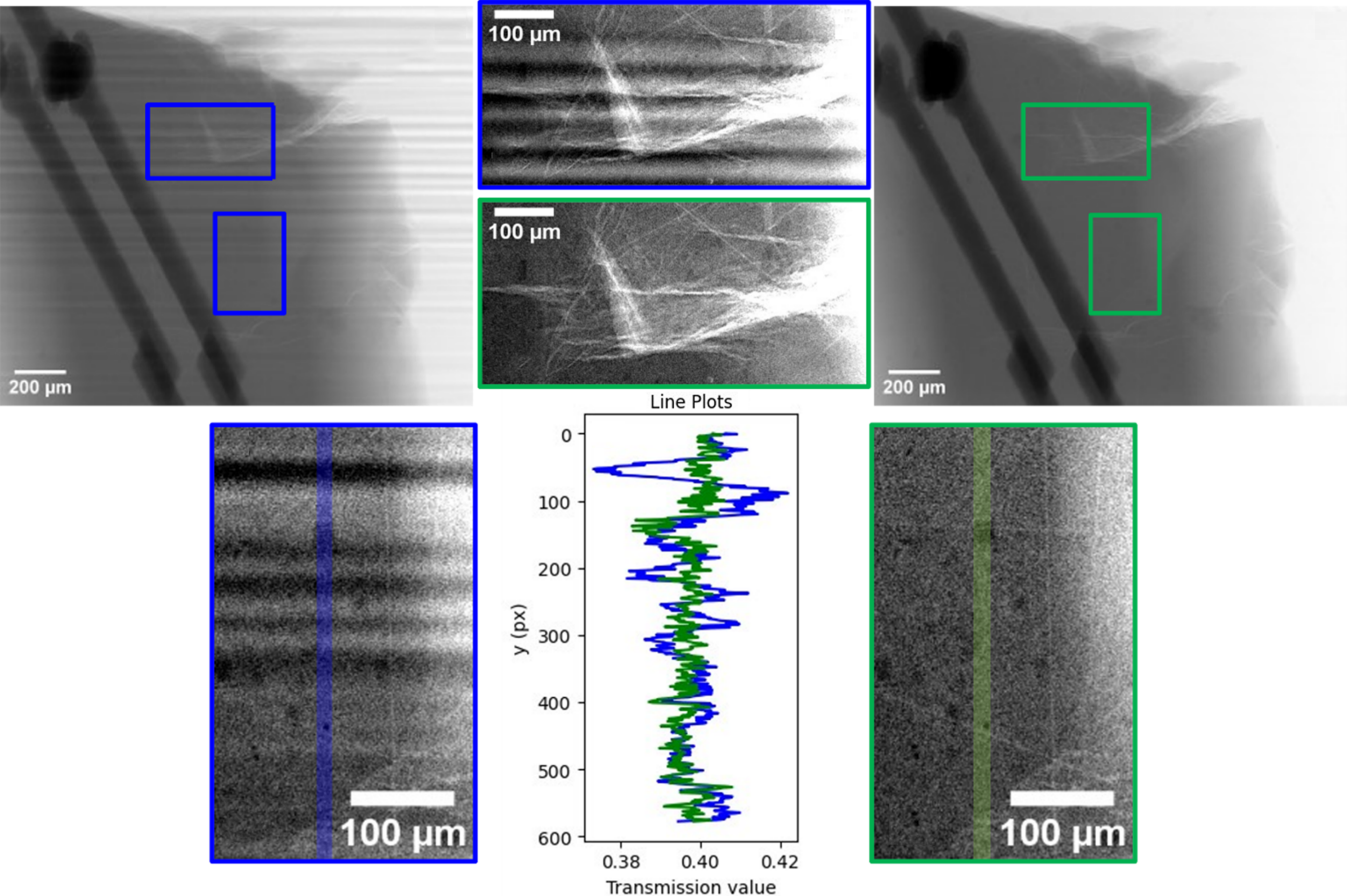
Radiographs of a daisy-chain structure, normalized using regular static flat-field correction (*a*, *c*, *e*) and multilayer-tilt normalization (*b*, *d*, *f*). 40 keV energy, 10× magnification (0.72 µm pixel size).

Figs. 7 ▸(*a*) and 7 ▸(*b*) show a radiograph of a sintered LTCC daisy chain, both normalized using static flat-field correction and our multilayer-tilt normalization technique. The multilayer-tilt corrected image shows very little presence of the DMM-induced stripe pattern, and a significantly better CNR than the static flat-field normalized image (13.2 against 10.8).

Figs. 7 ▸(*c*)–7 ▸(*f*) show different insets from the image, with Fig. 7 ▸(*g*) showing a vertical plot through the insets Figs. 7(*e*)–7(*f*). Darker particles in the ceramic, corresponding to alumina filler phases embedded in a brighter crystallized-glass matrix, are made much more visible in the *I*_DMM_ rather than the *I*_static−FF_ image.

## 4. Discussion

The comparison of both SNR and CNR between the two methods [static and dynamic correction (see Table 1 ▸)] showcases how efficient our technique is in attenuating the DMM-induced stripe pattern in the image and increasing contrast.

Our multilayer-tilt technique showcases a marked improvement in image quality, with the DMM-induced stripes effectively mitigated. This improvement is vital for downstream image analysis tasks (*e.g.* automated crack detection algorithms) where the overlap of stripes and actual sample cracks leads to false positives or missed detections.

**Figure 8:**
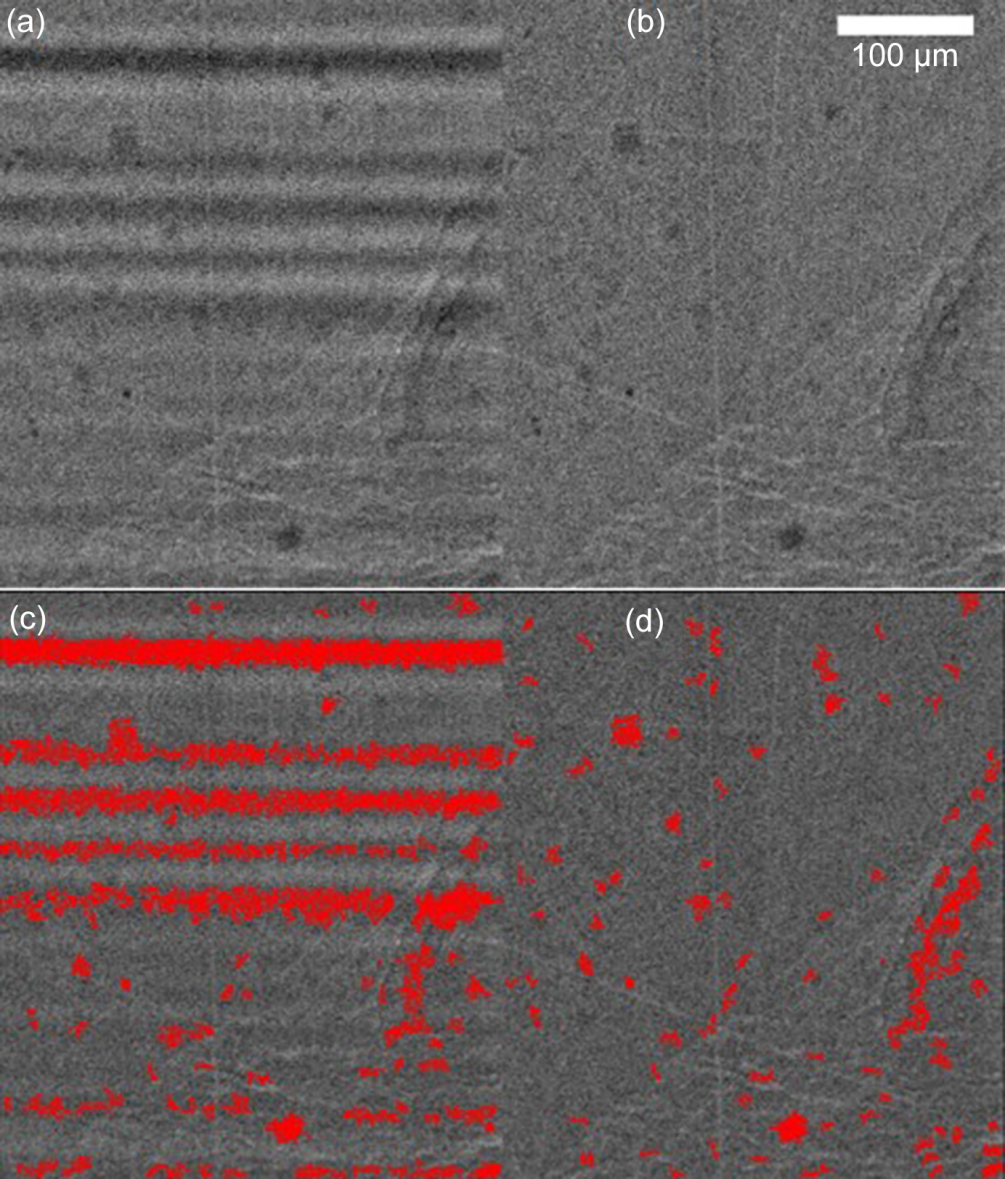
(*a*, *b*) Insets of Figs. 7 ▸(*a*) and 7 ▸(*b*), and (*c*, *d*) histogram threshold segmentation to find darker particles in the sintered ceramic, corresponding to Al_2_O_3_ granules.

Figs. 8 ▸(*a*) and 8 ▸(*b*) show insets of Figs. 7 ▸(*a*) and 7 ▸(*b*), respectively, which have been normalized using static flat-field normalization and DMM-tilt normalization.

Figs. 8 ▸(*c*) and 8 ▸(*d*) show the results of a particle segmentation on both images using *Fiji*. Both images have been sharpened using a Gaussian filter then segmented using histogram thresholding.

False positives found on the *I*_static−FF_ image are absent from the *I*_DMM_ image owing to the smoothing effect of the DMM stripe-pattern. Dark particles that were blended with dark DMM-induced stripes are now made visible and easily segmentable.

Another advantage of this technique is that it greatly reduces the time spent for flat-field acquisition, *e.g.* both the measurement 20× JIMA and the *ex situ* LTCC measurement were carried out by acquiring only one set of flat-fields in DMM-tilting mode at the beginning, which are then used for normalizing all subsequent projections. This flat-field acquisition is still essential, since DMM-induced stripes are not the only correction to be made (*e.g.* dust or scratches on the different windows or detector response must also be corrected).

At present, the shift compensation value is found manually through trial and error, and even though the computation is very fast, the normalization program could use automation to facilitate the end user’s experience. A starting shift value could be calculated via trigonometry using the DMM angle difference and the image quality metrics used above can be used as a loss function for automatic algorithms.

**Figure 9:**
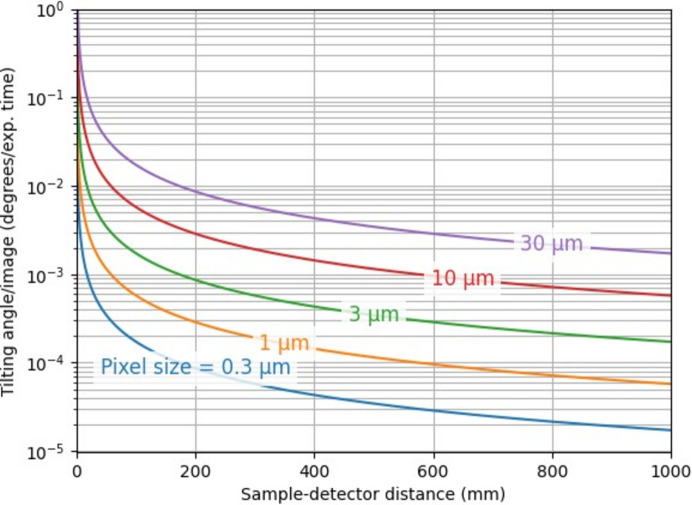
Contour plot of the maximum tilting angle per acquired image as a function of sample-to-detector distance, parameterized by the pixel size. The maximum speed is defined as the speed creating a vertical motion blur equal to half a pixel.

Fig. 9 ▸ shows a contour plot showcasing the maximum tilting angle speed available to the user relative to the distance between the sample and the detector and the pixel size chosen.

The DMM-tilting normalization technique requires the sample vertical shift between two consecutive images to be smaller than a pixel for the final image to be sharp. Otherwise, the motion-blur of the sample’s movement (not the stripes’) would cause information loss. All the plots in Fig. 9 ▸ show the maximum tilting angle per image to be used to avoid motion blur artifacts. The bigger the sample-to-detector distance and/or the smaller the pixel size, the slower the tilting needs to be.

This normalization technique is not limited to horizontal stripe artifacts caused by DMMs but could be applied to X-ray focusing mirrors, *e.g.* Kirkpatrick–Baez mirrors, which produce similar stripes.

Radiographic experiments requiring consistent and reliable detector response, free of DMM-induced artifacts would greatly benefit from this normalization technique. For instance, absorption edge radiography is a technique that consists in dividing radiograms taken below and above the X-ray energy edge of a given element present in the sample (Arlt *et al.*, 2013 ▸, 2019 ▸). Since the change in transmission is negligible in all materials except the one under study, the division of both radiograms provides an accurate map of the studied element.

## 5. Conclusions and outlook

DMMs provide large photon flux and are often used for *in situ*X-ray imaging of fast processes. Their use comes at the cost of the presence of horizontal dark-bright stripes all over the recorded image. These stripes, originating from thickness inhomogeneities, appear for every material coating choice, even for state-of-the-art multilayer depositions. The static flat-field correction is widely used to remove these stripes, but beam and thermal instabilities move the stripe pattern during experiment and require either frequent flat-field acquisition or the presence of stripes in the final image. Both solutions can hinder the study of fast processes and jeopardize quantitative image analysis (*e.g.* particle segmentation).

We introduced a novel approach for mitigating the DMM-induced stripe patterns in synchrotron X-ray radiography, based on the dynamic tilting of the DMM to dampen the presence of the stripes without compromising resolution.

The essential principle of our multilayer-tilting technique is to acquire projections during a small tilt of the second mirror of the DMM. We obtain a stack of projections that need to be vertically realigned before being averaged together. This alternative normalization method provides cleaner images without the presence of stripe pattern hindering image analysis and without compromising image resolution.

Future directions for this technique include the application to fast *in situ* processes and alternate methods of X-ray imaging, such as differential absorption-edge radiography.

The DMM-tilting normalization technique can be considered an alternative to overcome the presence of DMM-induced stripes and achieve stripe-free images.

## Figures and Tables

**Table 1:** SNR, STD and CNR of the tungsten material in the JIMA-target sample

Image	SNR (%)	STD Fig. 5 ▸(*c*)	CNR
*I*_static−FF_	18.3	0.00366	7.8
*I*_DMM_	28.4	0.00073	10.8

## References

[bb2] Arlt, T., Mahnke, H.-E., Siopi, T., Menei, E., Aibéo, C., Pausewein, R.-R., Reiche, I., Manke, I. & Lepper, V. (2019). *J. Cult. Herit.* **39**, 13–20.

[bb4] Arlt, T., Maier, W., Tötzke, C., Wannek, C., Markötter, H., Wieder, F., Banhart, J., Lehnert, W. & Manke, I. (2014). *J. Power Sources*, **246**, 290–298.

[bb3] Arlt, T., Manke, I., Wippermann, K., Riesemeier, H., Mergel, J. & Banhart, J. (2013). *J. Power Sources*, **221**, 210–216.

[bb1] Arlt, T., Schröder, A., Heyne, K., Riesemeier, H., Wippermann, K., Lehnert, W. & Manke, I. (2015). *J. Power Sources*, **297**, 83–89.

[bb5] Bandyopadhyay, A. & Heer, B. (2018). *Mater. Sci. Eng. Rep.* **129**, 1–16.

[bb6] Bangsgaard, K. O., Burca, G., Ametova, E., Andersen, M. S. & Jørgensen, J. S. (2023). *Appl. Math. Sci. Eng.* **31**, 2176000.

[bb7] Capraro, B., Heidenreich, M. & Töpfer, J. (2022). *Materials*, **15**, 564 10.3390/ma15020564 PMC8778247 35057282

[bb8] Feller, C. & Partsch, U. (2021). *J. Sens. Sens. Syst.* **10**, 83–91.

[bb9] Grigorev, A. Yu. & Buzmakov, A. V. (2023). *Bull. Russ. Acad. Sci. Phys.* **87**, 604–610.

[bb10] Haussmann, J., Markötter, H., Alink, R., Bauder, A., Dittmann, K., Manke, I. & Scholta, J. (2013). *J. Power Sources*, **239**, 611–622.

[bb11] Havariyoun, G., Massimi, L., Hagen, C., Endrizzi, M. & Olivo, A. (2023). *Phys. Med. Biol.* **68**, 09NT02 10.1088/1361-6560/acc927 36996845

[bb12] Hu, L., Wang, H., Sutter, J. P. & Sawhney, K. (2021). *Opt. Express*, **29**, 4270. 10.1364/OE.417030 33771010

[bb13] Hubert, M., Pacureanu, A., Guilloud, C., Yang, Y., da Silva, J. C., Laurencin, J., Lefebvre-Joud, F. & Cloetens, P. (2018). *Appl. Phys. Lett.* **112**, 203704.

[bb14] Isaac, A., Antunes, F. A. F., Conti, R., Montoro, L. A., Malachias, A., Massara, P., Kitten, G., Markötter, H., Manke, I. & Silva, S. S. (2018). *Ind. Crops Prod.* **114**, 19–27.

[bb15] Isaac, A., Conti, R., Viana, C. M., Sket, F. I., Montoro, L. A., Hilger, A. & Manke, I. (2016). *Ind. Crops Prod.* **86**, 289–294.

[bb17] Kastengren, A., Powell, C. F., Arms, D., Dufresne, E. M., Gibson, H. & Wang, J. (2012). *J. Synchrotron Rad.* **19**, 654–657. 10.1107/S0909049512016883 PMC3579593 22713903

[bb18] Markötter, H., Sintschuk, M., Britzke, R., Dayani, S. & Bruno, G. (2022). *J. Synchrotron Rad.* **29**, 1292–1298. 10.1107/S1600577522007342 PMC9455212 36073889

[bb31] Matz, R., Rabe, T., Töpfer, J. & Ziesche, S. (2020). *J. Ceram. Sci. Technol.* **11**, 44–61.

[bb19] Morawe, C. (2019). *AIP Conf. Proc.* **2054**, 060002.

[bb20] Morawe, C., Carau, D. & Peffen, J.-C. (2017). *Proc. SPIE*, **10386**, 1038603.

[bb22] Quispe, M., Garriga, D., González, N., Šics, I., Colldelram, C., Nicolas, J. & Juanhuix, J. (2022). *J. Phys. Conf. Ser.* **2380**, 012073.

[bb23] Rack, A., Morawe, C., Mancini, L., Dreossi, D., Parkinson, D. Y., MacDowell, A. A., Siewert, F., Rack, T., Holz, T., Krämer, M. & Dietsch, R. (2014). *Proc. SPIE*, **9207**, 92070V.

[bb24] Rack, A., Weitkamp, T., Riotte, M., Rack, T., Dietsch, R., Holz, T., Krämer, M., Siewert, F., Meduna, M., Morawe, C., Cloetens, P. & Ziegler, E. (2010). *Proc. SPIE*, **7802**, 78020M. 10.1107/S0909049510011623 20567082

[bb25] Schindelin, J., Arganda-Carreras, I., Frise, E., Kaynig, V., Longair, M., Pietzsch, T., Preibisch, S., Rueden, C., Saalfeld, S., Schmid, B., Tinevez, J.-Y., White, D. J., Hartenstein, V., Eliceiri, K., Tomancak, P. & Cardona, A. (2012). *Nat. Methods*, **9**, 676–682. 10.1038/nmeth.2019 PMC3855844 22743772

[bb26] Soares, A. P., Baum, D., Hesse, B., Kupsch, A., Müller, B. R. & Zaslansky, P. (2021). *Dent. Mater.* **37**, 201–211. 10.1016/j.dental.2020.10.018 33317826

[bb27] Stampanoni, M., Groso, A., Isenegger, A., Mikuljan, G., Chen, Q., Meister, D., Lange, M., Betemps, R., Henein, S. & Abela, R. (2007). *AIP Conf. Proc.* **879**, 848–851.

[bb28] Tötzke, C., Gaiselmann, G., Osenberg, M., Bohner, J., Arlt, T., Markötter, H., Hilger, A., Wieder, F., Kupsch, A., Müller, B. R., Hentschel, M. P., Banhart, J., Schmidt, V., Lehnert, W. & Manke, I. (2014). *J. Power Sources*, **253**, 123–131.

[bb29] Zehbe, R. H., Riesemeier, H., Kirkpatrick, C. J. & Brochhausen, C. (2012). *Micron*, **43**, 1060–1067. 10.1016/j.micron.2012.05.001 22633854

[bb30] Zhao, L., Liu, F., Shen, X., Jing, G., Cai, Y.-M. & Li, Y. (2018). *IEEE Access*, **6**, 38097–38105.

